# Curando Corações com Humor: O Potencial da Risoterapia na Reabilitação Cardíaca

**DOI:** 10.36660/abc.20240594

**Published:** 2024-11-27

**Authors:** Cleidiane Andrade, Filipe Ferrari, Ricardo Stein

**Affiliations:** 1 Universidade Federal do Rio Grande do Sul Faculdade de Medicina Porto Alegre RS Brasil Programa de Pós-Graduação em Cardiologia e Ciências Cardiovasculares – Faculdade de Medicina – Universidade Federal do Rio Grande do Sul, Porto Alegre, RS – Brasil; 2 Hospital de Clínicas de Porto Alegre Grupo de Pesquisa em Cardiologia do Exercício Porto Alegre RS Brasil Grupo de Pesquisa em Cardiologia do Exercício (CardioEx) – Hospital de Clínicas de Porto Alegre, Porto Alegre, RS – Brasil; 3 Universidade Federal do Rio Grande do Sul Departamento de Medicina Interna Porto Alegre RS Brasil Departamento de Medicina Interna – Universidade Federal do Rio Grande do Sul, Porto Alegre, RS – Brasil

**Keywords:** Risoterapia, Doença Arterial Coronariana, Brasil

## Introdução

A risoterapia, também conhecida como terapia do humor, utiliza o riso e o prazer como ferramentas para melhorar o bem-estar físico e emocional. Esta prática oferece inúmeros benefícios, incluindo: (1) redução dos níveis de cortisol,^1^ reduzindo assim o estresse – um fator de risco cardiovascular bem estabelecido; (2) melhora da circulação sanguínea através da liberação de substâncias vasoativas;^2^ e (3) melhora do humor, o que ajuda a combater a depressão e a ansiedade^3^ – condições comumente encontradas em pacientes com doenças cardiovasculares e que também são fatores de risco para o desenvolvimento de doença arterial coronariana (DAC).^4,5^

Um estudo conduzido nos EUA envolvendo 48 pacientes diabéticos que tinham sofrido recentemente um infarto do miocárdio dividiu esses participantes em dois grupos.^6^ Um grupo recebeu reabilitação cardíaca padrão, além de sessões diárias de humor de 30 minutos de sua escolha (intervenção), enquanto o outro grupo recebeu apenas reabilitação padrão (controle). As descobertas revelaram que o grupo de humor apresentou menos arritmias, níveis reduzidos de epinefrina e norepinefrina na urina e no plasma, uma necessidade reduzida de nitroglicerina para controlar a angina e menos infartos recorrentes.^6^ Além disso, Miller et al.^7^ demonstraram que assistir a um filme de comédia aumenta significativamente a dilatação fluxo-mediada em indivíduos saudáveis, enquanto assistir a filmes estressantes tende a reduzir essa dilatação. Além disso, um estudo aberto com 17 participantes idosos que assistiram a comédia stand-up uma vez por semana durante 4 semanas relatou reduções na frequência cardíaca e na pressão arterial, juntamente com aumento nos níveis plasmáticos de serotonina.^8^ Outras evidências sugerem que pacientes com doenças cardiovasculares são menos propensos a rir muito.^9^ Além disso, a prevalência de doenças cardíacas entre idosos é 1,2 vezes maior naqueles que nunca riem em comparação com aqueles que riem diariamente.^10^

Coletivamente, essas descobertas destacam o potencial do humor como uma estratégia eficaz para melhorar a saúde cardiovascular e a qualidade de vida geral.

### Risoterapia em pacientes com doença arterial coronariana

O imperativo de melhorar os resultados globais de saúde para pacientes com DAC merece nossa atenção e deve ser priorizado por várias razões. Primeiro, apesar dos avanços significativos no tratamento, a DAC continua sendo a principal causa de morte no mundo.^11^ Em segundo lugar, pacientes que apresentam baixa qualidade de vida relacionada à saúde de um a três anos após um infarto do miocárdio podem estar sob risco aumentado de eventos adversos, incluindo morte.^12^ Pesquisas contínuas que visam melhorar o prognóstico de indivíduos que sofreram de síndrome coronariana aguda são um foco crítico na área.

Curiosamente, a maioria dos estudos sobre risoterapia se concentrou em seus efeitos de curto prazo em variáveis cardiovasculares, principalmente em indivíduos saudáveis ou aqueles com condições diferentes de DAC. Há informações limitadas sobre como a risoterapia afeta pacientes com DAC. Até onde sabemos, nenhum ensaio clínico randomizado foi conduzido no Brasil para avaliar essa terapia nessa população de pacientes. No entanto, dados de um estudo anterior conduzido por nosso grupo de pesquisa (CardioEx) no Hospital de Clínicas de Porto Alegre indicaram que uma única sessão de comédia de 30 minutos melhorou significativamente os parâmetros hemodinâmicos em pacientes com DAC estável.^13^ Os participantes que assistiram a um filme de comédia (com média de 63 ± 31 risadas genuínas) apresentaram maior volume sistólico máximo (21,2 ml; 24,8%) e débito cardíaco (1,6 L/min; 27,1%) em comparação com aqueles que assistiram a um documentário neutro (p < 0,05). No entanto, análises de longo prazo são necessárias, principalmente com foco em parâmetros mais relevantes no contexto da doença cardiovascular.

Temos o prazer de compartilhar que estamos atualmente conduzindo um ensaio clínico randomizado para preencher essa lacuna. Nosso grupo de pesquisa está investigando os efeitos da risoterapia na capacidade funcional, função endotelial e biomarcadores inflamatórios em pacientes com DAC estável (idade média [DP] de 64 ± 10 anos). Os participantes foram designados para um grupo de intervenção, que assistiu a 30 minutos de um filme de comédia, ou um grupo de controle, que assistiu a 30 minutos de um documentário neutro. Análises interinas (dados não publicados) indicam que 24 sessões de risoterapia podem melhorar significativamente o VO_2_pico. Ainda mais encorajador, após 3 meses de risoterapia, o VO_2_pico aumentou em 11%, um resultado comparável aos vistos em alguns programas de reabilitação cardíaca focados em exercícios.

Vale sempre a pena enfatizar que melhorar a capacidade de exercício é um objetivo fundamental da reabilitação cardíaca. Mais de duas décadas atrás, Myers et al.^14^ estabeleceram que a capacidade de exercício é um preditor mais forte de mortalidade em homens do que muitos outros fatores de risco cardiovascular bem conhecidos. Especificamente, o aumento do VO_2_pico demonstrou fornecer benefícios significativos na redução da carga de DAC^15^ e é um forte preditor de futuras readmissões cardiovasculares e mortalidade por todas as causas.^16^

## Conclusões

Até onde sabemos, nosso estudo é o primeiro ensaio clínico randomizado a avaliar o VO_2_pico de longo prazo após a risoterapia em indivíduos com DAC estável. Embora os desafios de melhorar o prognóstico para pacientes com doenças cardiovasculares sejam inegáveis, é encorajador ver um foco crescente em abordagens multifacetadas para lidar com essa questão.

Além disso, não é brincadeira – entre essas novas abordagens parece estar a risoterapia.
