# Supplementary material for: Curando Corações com Humor: O Potencial da Risoterapia na Reabilitação Cardíaca
Source: Arq Bras Cardiol. 2024 Nov 27;121(11):e20240594. [Article in Portuguese] doi: 10.36660/abc.20240594 (PMC11634290; doi:10.36660/abc.20240594)
Supplement: Translation

# Translation: Healing Hearts with Humor: The Potential of Laughter Therapy in Cardiac Rehabilitation

**DOI:** 10.36660/abc.20240594i

**Authors:** Cleidiane Andrade, Filipe Ferrari, Ricardo Stein

**Affiliations:** 1 Porto Alegre RS Brazil Programa de Pós-Graduação em Cardiologia e Ciências Cardiovasculares – Faculdade de Medicina – Universidade Federal do Rio Grande do Sul, Porto Alegre, RS – Brazil; 2 Porto Alegre RS Brazil Grupo de Pesquisa em Cardiologia do Exercício (CardioEx) – Hospital de Clínicas de Porto Alegre, Porto Alegre, RS – Brazil; 3 Porto Alegre RS Brazil Departamento de Medicina Interna – Universidade Federal do Rio Grande do Sul, Porto Alegre, RS – Brazil

**Keywords:** Laughter Therapy, Coronary Artery Disease, Brazil

## Introduction

Laughter therapy, also known as humor therapy, utilizes laughter and enjoyment as tools to enhance physical and emotional well-being. This practice offers numerous benefits, including: (1) lowering cortisol levels,^1^ thereby reducing stress – a well-established cardiovascular risk factor; (2) improving blood circulation through the release of vasoactive substances;^2^ and (3) boosting mood, which helps to combat depression and anxiety^3^ – conditions commonly found in patients with cardiovascular diseases and which are also risk factors for the development of coronary artery disease (CAD).^4,5^

A study conducted in the USA involving 48 diabetic patients who had recently experienced a myocardial infarction divided these participants into two groups.^6^ One group received standard cardiac rehabilitation in addition to 30-minute daily humor sessions of their choice (intervention), while the other group received only standard rehabilitation (control). The findings revealed that the humor group experienced fewer arrhythmias, reduced levels of epinephrine and norepinephrine in both urine and plasma, a decreased need for nitroglycerin to manage angina, and fewer recurrent infarctions.^6^ Additionally, Miller et al.^7^ demonstrated that watching a comedy film significantly increases flow-mediated dilation in healthy individuals, whereas viewing stressful films tends to reduce this dilation. Furthermore, an open-label trial with 17 elderly participants who watched stand-up comedy once a week for 4 weeks reported reductions in heart rate and blood pressure, along with increased plasma serotonin levels.^8^ Further evidence suggests that patients with cardiovascular disease are less likely to laugh heartily.^9^ Moreover, the prevalence of heart disease among the elderly is 1.2 times higher in those who never laugh compared to those who laugh daily.^10^

Collectively, these findings highlight the potential of humor as an effective strategy for improving cardiovascular health and overall quality of life.

### Laughter therapy in patients with coronary artery disease

The imperative to improve global health outcomes for patients with CAD deserves our attention and should be prioritized for several reasons. First, despite significant advancements in treatment, CAD remains the leading cause of death worldwide.^11^ Second, patients who experience low health-related quality of life one to three years after a myocardial infarction may be at increased risk of adverse events, including death.^12^ Ongoing research aimed at improving the prognosis of individuals who have suffered from acute coronary syndrome is a critical focus in the field.

Interestingly, most studies on laughter therapy have focused on its short-term effects on cardiovascular variables, primarily in healthy individuals or those with conditions other than CAD. There is limited information on how laughter therapy impacts patients with CAD. To our knowledge, no randomized clinical trials have been conducted in Brazil to evaluate this therapy in this patient population. However, data from a previous study conducted by our research group (CardioEx) at the Hospital de Clínicas de Porto Alegre indicated that a single 30-minute comedy session significantly improved hemodynamic parameters in patients with stable CAD.^13^ Participants who watched a comedy film (averaging 63 ± 31 genuine laughs) showed higher maximum stroke volume (21.2 ml; 24.8%) and cardiac output (1.6 L/min; 27.1%) compared to those who watched a neutral documentary (p < 0.05). Nonetheless, long-term analyses are necessary, particularly focusing on parameters that are more relevant in the context of cardiovascular disease.

We are pleased to share that we are currently conducting a randomized clinical trial to fill this gap. Our research group is investigating the effects of laughter therapy on functional capacity, endothelial function, and inflammatory biomarkers in patients with stable CAD (mean [SD] age 64 ± 10 years). Participants were assigned to either an intervention group, which watched 30 minutes of a comedy film, or a control group, which watched 30 minutes of a neutral documentary. Interim analyses (unpublished data) indicate that 24 sessions of laughter therapy can significantly improve VO_2_peak. Even more encouraging, after 3 months of laughter therapy, VO_2_peak increased by 11%, a result comparable to those seen in some cardiac rehabilitation programs focused on exercise.

It is always worth emphasizing that improving exercise capacity is a key goal of cardiac rehabilitation. More than two decades ago, Myers et al.^14^ established that exercise capacity is a stronger predictor of mortality in men than many other well-known cardiovascular risk factors. Specifically, increasing VO_2_peak has been shown to provide significant benefits in reducing the burden of CAD^15^ and is a strong predictor of future cardiovascular readmissions and all-cause mortality.^16^

## Conclusions

To the best of our knowledge, our study is the first randomized clinical trial to evaluate long-term VO_2_peak following laughter therapy in individuals with stable CAD. While the challenges of improving the prognosis for cardiovascular disease patients are undeniable, it is encouraging to see an increasing focus on multifaceted approaches to tackle this issue.

Furthermore, it is no joke – among these new approaches appears to be laughter therapy.
